# Shedding of the Pandemic Swine-Origin Influenza A Virus (H1N1) after Oseltamivir Administration

**DOI:** 10.1155/2010/976084

**Published:** 2010-12-14

**Authors:** Leiyun Weng, Qiang Wang, Wei Wang, Peijun Ren, Vincent Deubel, Tetsuya Toyoda

**Affiliations:** ^1^Unit of Viral Genome Regulation, Institut Pasteur of Shanghai, The Key Laboratory of Virology and Immunology, Chinese Academy of Sciences, 411 Hefei Road, Shanghai 200025, China; ^2^Emerging Infectious Diseases, Institut Pasteur of Shanghai, The Key Laboratory of Virology and Immunology, Chinese Academy of Sciences, 411 Hefei Road, Shanghai 200025, China; ^3^Institut Pasteur in Cambodia, 5 Monivong Boulevard, P.O. Box 983, Phnom Penh, Cambodia; ^4^Choju Medical Institute, Fukushimura Hospital, 19-4 Azanakayama, Noyori-cho, Toyohashi, Aichi 441-8124, Japan; ^5^Infectious Disease Regulation Project, Tokyo Metropolitan Institute of Medical Sciences, 1-6, Kamikitazawa 2-chome, Setagaya-ku, Tokyo156-8506, Japan

## Abstract

We analyzed the virus shedding of an oseltamivir-treated patient who had been infected with the pandemic swine-origin influenza A (H1N1) virus which had an oseltamivir-sensitive neuraminidase. The virus was isolated from the pharyngeal swabs of the patient using MDCK cells, and the virus genome RNA was detected in the same samples both by real-time RT-PCR and RT-PCR. The virus was isolated until 44 h after oseltamivir administration although the virus genome was detected until one day after oseltamivir treatment was stopped. Due to their high sensitivity, RT-PCR and real-time RT-PCR may cause misdiagnosis by detection of viral genome which does not infect, and classical virus isolation and clinical symptoms are recommended for the evaluation of oseltamivir treatment.

## 1. Introduction

The novel swine origin influenza A virus (S-OIV) (H1N1) caused pandemic in 2009-2010 [1]. Oseltamivir (Tamiflu, Hoffmann LaRoche) is recommended for the treatment of S-OIV infection because it is naturally resistant to amantadine and rimantadine but is susceptible to the neuraminidase inhibitors, oseltamivir, and zanamivir (Relenza, Glaxo Smithkline) although the oseltamivir-resistant neuraminidase (NA) H275Y (N1 numbering) was detected in some isolates [2–4]. Here, we precisely report the virus shedding of the pandemic S-OIV after oseltamivir treatment (Table 1).

## 2. Case Presentation

A 24-year-old female in Shanghai, China was infected with the pandemic S-OIV in September 2009. She had received a seasonal flu vaccination (Sanofi Pasteur) the previous year. 

Her first symptom was a sore throat, and on the 4th day after the onset of her sore throat, she had a high fever (>38.5°C). The next day (the 5th day after onset), she visited a clinic. Her blood cell count was normal. Her chest X-ray did not show bronchitis or pneumonia. Oseltamivir (75 mg × 2) was administered at 36 hours after the onset of her high fever for five days. On the second day of oseltamivir administration, her high fever disappeared, and all eight segments of the S-OIV genome were cloned from her pharyngeal swabs, and the sequences of the pandemic S-OIV were confirmed. Its neuraminidase (NA) contained an oseltamivir-sensitive sequence (275H). Since then, pharyngeal swabs were collected everyday for eight days and subjected to virus isolation and examined for virus genome RNA (from the 6th to the 13th day). 

Each pharyngeal swab was aliquoted into three fractions: for virus isolation, for RT-PCR, and for real-time RT-PCR, and each experiment was performed in a different lab. For virus genome detection, RNA was extracted from the pharyngeal swabs using Trizol (Invitrogen). The RNA was then analyzed by real time RT-PCR for S-OIV haemagglutinin (HA), which was performed in a SmartCycler II System (Cepheid) using a QuantiTect Probe RT-PCR kit (Qiagen) with the primers (HA primer 1 and 2) and HA probe listed in Table 2. At the same time, the RNA was analyzed for the eight genome segments by RT-PCR. The cDNA was reverse transcribed by MMLV RTase (Takara) using Uni12 (AGCAAAGCAGG and AGCGAAAGCAGG) [5], before being subjected to PCR using LA-Taq (Takara) for the coding frames of the eight genome segments and the primers listed in Table 2. 

Her pharyngeal swabs were also inoculated into MDCK cells after filtration through 0.22 *μ*m filter (Millipore) and then incubated in DMEM with 2 *μ*g/ml trypsin (DIFCO), 1 mg/ml BSA (Sigma), 50 units/ml of penicillin (Invitrogen), 50 *μ*g/ml of streptomycin (Invitrogen), and 0.1 mg/ml kanamycin (Invitrogen) at 37°C for the detection of cytopathic effects (CPEs). When CPEs were detected, the pandemic S-OIV genome segments were also identified by RT-PCR of the culture supernatant.

Oseltamivir is accepted as the standard treatment for S-OIV [2, 4]. Although oseltamivir was effective, the virus was isolated from her pharyngeal swabs on the second (20 hours) and third (44 hours) days of oseltamivir administration. The eight viral genome segments were detected by RT-PCR, and HA genome was detected by real-time RT-PCR until the day after the end of oseltamivir administration. No other people around her showed any symptoms. 

By 36 hours of oseltamivir administration in previous cases, the influenza A/Texas/91 (H1N1) virus had disappeared from the patients' nasopharyngeal swabs [6]. In this case, the virus (influenza virus A/Shanghai/P1/2009 (H1N1)) was still isolated from the patient's pharyngeal swab on the third day (44 hours) of oseltamivir administration, and the viral genome segments were detected until the day after oseltamivir administration was stopped. RT-PCR detection is more sensitive than virus isolation [7]. The +++ and ++ scores of real-time RT-PCR correlated with those of virus isolation. The prolonged detection of viral RNA without the oseltamivir-resistant mutation of NA by RT-PCR or real-time RT-PCR from the S-OIV-infected patients administrated with oseltamivir was also reported previously [8, 9]. All of these reports indicate that S-OIV could persist in the patients although shedding of S-OIV may vary from case to case. However, due to their high sensitivity, RT-PCR and real-time RT-PCR may cause misdiagnosis, and the classical virus isolation and clinical symptoms are still recommended [10].

## 3. Conclusions

The classical virus isolation and clinical symptoms are recommended for the evaluation of oseltamivir treatment although RT-PCR and real-time RT-PCR are convenient.

##  Consent 

Written informed consent was obtained from the patient for publication of this case report and any accompanying images. A copy of the written consent is available for review by the Editor-in-Chief of this journal.

##  Conflict of Interests 

The authors declare that they have no conflict of interests.

##  Authors' Contributions 

L. Weng and Q. Wang collected samples and performed RT-PCR, W. Wang, P. Ren, and V. Deubel performed virus isolation and real-time RT-PCR, and L. Weng and T. Toyoda wrote the paper.

## Figures and Tables

**Table 1:** Symptoms and detection of S-OIV.

Date (2009)	Sep 16	17	18	19	20	21	22	23	24	25	26	27	28
Time (day)	1	2	3	4	5	6	7	8	9	10	11	12	13

Oseltamivir					+^1^	++	++	++	++	+			
Real-time RT-PCR						+++	++	+	+	+	+	−	−
RT-PCR						+	+	+	+	+	+	−	−
Virus isolation						+^2^	+^3^	−	−	−	−	−	−
Fever (>38.5°C)			−	+	+	−	−	−	−	−	−	−	
Sore throat	+	+	+	+	+	+++	+++	+++	++	−	−	−	
Headache	−	−	+	+	++	++	+++	+++	+	−	−	−	
Cough	−	−	+	+	+	+++	+++	+++	++	+	±	−	

^1^Seventy-five mg of oseltamivir. The first tablet was administered around 21:00.

^2^Pharyngeal swab was taken around 17:00 (20 h after oseltamivir administration).

^3^Pharyngeal swab was taken around 17:00 (44 h after oseltamivir administration).

**Table 2:** Sequences used for RT-PCR and real-time RT-PCR.

Name	Sequence (5′→3′)
PB2 f (BamHI)	CTGGATCCATGGAGAGAATAAAAGAACTGAGAGATC
PB2 r (NotI)	CTGCGGCCGCCTAATTGATGGCCATCCGAATTC
PB1 f (BamHI)	CTGGATCCATGGATGTCAATCCGACTCTACTTTTC
PB1 r (NotI)	CTGCGGCCGCTTATTTTTGCCGTCTGAGTTCTTC
PA f (BamHI)	GCGGATCCATGGAAGACTTTGTGCGACAATG
PA r (NotI)	GCGCGGCCGCCTACTTCAGTGCATGTGTGAGGAAGG
HA f (NheI)	GCTAGCCATGAAGGCAATACTAGTAGTTCTGC
HA r (EcoRI)	GAATTCTTAAATACATATTCTACACTGTAGAGACCC
NP f (KpnI)	GCGGTACCATGGCGTCTCAAGGCACCA
NP r (PstI)	ATCTGCAGTCAACTGTCATACTCCTCTGCATTGTC
NA f	ATGAATCCAAACCAAAAGATAATAACC
NA r	TTACTTGTCAATGGTAAATGGCAAC
M f	ATGAGTCTTCTAACCGAGGTCG
M r	TTACTCTAGCTCTATGTTGACAAAATG
NS f	ATGGACTCCAACACCATGTCAAG
NS r	TTAAATAAGCTGAAACGAGAAAGCTCT
HA primer 1	GTAAAGCTGGAATCAACAAGG
HA primer 2	GAGACCCATTAGAGCACATC
HA probe	TET-GATTGCCCCCAGGGAGACTA-BHQ1

## List of Abbreviations

WHO: The World Health Organization
S-OIV: The novel swine origin influenza A virus
HA: Haemagglutinin
NA: Neuraminidase
RT-PCR: Reverse transcription polymerase chain reaction
CPE: Cytopathic effect.
